# Augmented Reality in Interventional Radiology: A Review of Systems, Ergonomics, Applications, and Outcomes

**DOI:** 10.1177/29941520251377069

**Published:** 2025-09-19

**Authors:** Youssef Ghosn, Javin Schefflein, Yara Jabbour, Debkumar Sarkar, Francois H. Cornelis

**Affiliations:** ^1^ Department of Radiology, Memorial Sloan Kettering Cancer Center, New York, NY, USA.; ^2^ Department of Radiology, American University of Beirut Medical Center, Riad El Solh, Beirut, Lebanon.; ^3^ Department of Radiology, Weill Cornell Medical College, New York, NY, USA.

**Keywords:** interventional radiology, interventional oncology, augmented reality, mixed reality, virtual reality

## Abstract

Interventional radiology (IR) procedures traditionally rely on two-dimensional imaging for guidance. Augmented reality (AR) offers a transformative approach by overlaying two-dimensional radiological images or three-dimensional digital reconstructions onto the real-world environment, potentially improving procedural precision and operator ergonomics. This narrative literature review examines AR systems, procedural workflow, operator ergonomics, current clinical applications, and outcomes. Inclusion criteria include studies investigating AR in IR, excluding articles unrelated to IR procedures. Current literature suggests improved ergonomics, mainly enhanced depth perception, decreased eye movement/fatigue, and decreased cybersickness with overall operator tolerability. Clinical studies demonstrated reductions in radiation exposure and procedural times, with comparable accuracy to standard techniques. Limitations include the lack of large-scale clinical data and standardized evaluation metrics.

## Introduction

Interventional radiology (IR) relies on imaging guidance to navigate anatomical structures and perform interventions with high accuracy. Traditional approaches demand that operators mentally reconstruct three-dimensional (3D) relationships from two-dimensional (2D) imaging, thereby increasing cognitive and physical strain while limiting certain procedures’ scope.^1–3^ In this context, extended reality (XR) technologies have emerged as promising tools to address the limitations of a traditional IR approach.^4–6^

XR is an umbrella term encompassing virtual reality (VR), augmented reality (AR), and mixed reality (MR). VR allows for the creation of entirely immersive digital environments that replace the real world—facilitating radiology procedure simulation, intervention planning, and skill refinement in a risk-free setting. AR superimposes digital data, such as preoperative 3D reconstructed magnetic resonance and computed tomography images, onto the physical world, thereby enabling radiologists to visualize holographic overlays and monitor their position relative to anatomical structures in real-time. MR combines digital and natural elements to create an environment where the user can simultaneously interact with both the virtual and real worlds.^1,2,7^ Because the terms AR and MR are used interchangeably in the literature, this review adopts the term AR exclusively.^7^

An interactive AR environment requires the orchestration of various components, including sensors, proper AR software, and display devices.^8^ Together, these technologies aim to optimize the precision of interventional procedures, potentially improving patient outcomes by enhancing procedural visualization, navigation, and overall workflow in the IR suite. Further, because procedural specialties such as IR have a high prevalence of work-related musculoskeletal disorders,^3,9^ AR introduces a new dimension of ergonomic support, potentially reducing occupational strain. Challenges hindering the wide adoption of AR in IR include potential costs and equipment-specific limitations.^1,2^ Additionally, the evolution of AR has the potential to result in increased reliance on holograms, reducing the need for real-time image guidance and potentially disrupting IR as a specialty. This narrative literature review aims to provide an overview of AR applications in IR, focusing on system descriptions, workflows, and ergonomics, as well as current applications and outcomes.

## Methodology

Relevant articles were selected via review of available literature following PROSPERO guidelines and the PRISMA 2020 checklist.^10,11^ The search was undertaken through PubMed, Scopus, Cochrane Library, Embase, Web of Science, and Google Scholar. Additional relevant articles were identified through references cited in key reviews and original studies. The search spanned publications from January 2005 to October 2024. Keywords included “extended reality”, “augmented reality”, “virtual reality”, “mixed reality”, “interventional radiology”, “interventional oncology”, “AR/VR in image-guided interventions,” “navigation systems in interventional radiology,” and “ergonomics in interventional radiology.” Boolean operators and truncation were applied to refine the search. Inclusion criteria comprised studies discussing AR applications in IR, types of navigation systems, role in intraprocedural planning and guidance, effect on workflow and ergonomics, and impacts on procedural outcomes, including radiation dose, procedural speed, accuracy, and other outcomes. Articles not in English and those focused on different medical fields were excluded.

**FIG. 1.**
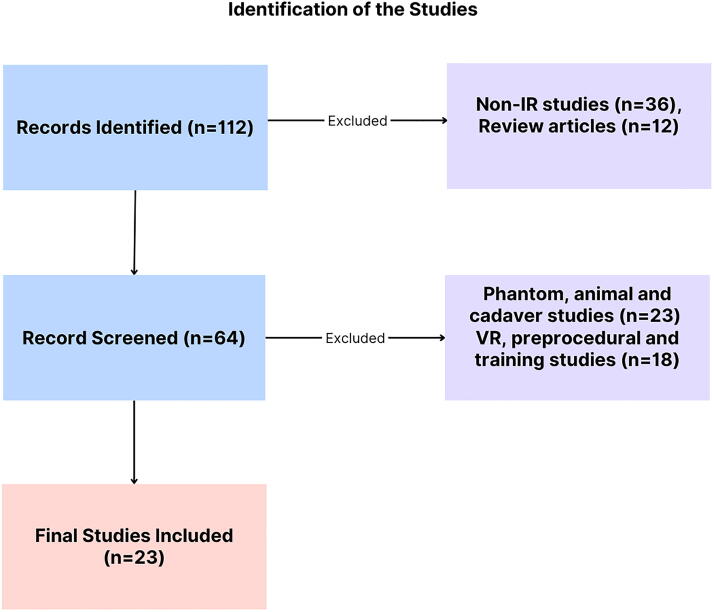
Flowchart of research selection for the “Applications and Outcomes” section.

Additional parameters were applied for the “Applications and Outcomes” section. Research done strictly on phantoms, animals, or cadavers was excluded. Those focused on the use of VR alone, or the use of AR strictly for simulation training, pre-procedural planning, patient education purposes, or telemedicine were also excluded. Selected studies were evaluated individually, and the following variables were sought for data collection: year of study, AR system used, type of display used, type of procedure, type of image guidance used, participants, study design, and results. The methodological quality of the included studies was evaluated using established tools tailored to study design and employing the Newcastle-Ottawa Scale for cohort studies.^12^ If a study did not explicitly indicate a particular study design, the latter was inferred during evaluation. Figure 1 presents a flowchart depicting study selection for the “Applications and Outcomes” section (Fig. 1).

All stages of the literature search, article screening, and data extraction were conducted by a single reviewer. A second reviewer verified the selected studies to improve reliability and minimize potential bias.

## Results

### Systems description

#### System overview

The software and hardware for AR visualization in image-guided intervention environments vary, but generally include a frame grabber, an image processing unit (such as a computer with specific software), a data transfer network, and a direct AR display (such as head-mounted display [HMD]). Other components include various sensors for tracking and registration.^13^ The frame grabber (not always needed) is hardware that is connected to an output port of an imaging source and has access to the imaging data. The image processing framework is responsible for converting, scaling, enhancing, and encoding the image at runtime. The data transfer network sends the “processed images” from the image processing framework to an HMD.^13^

**FIG. 2.**
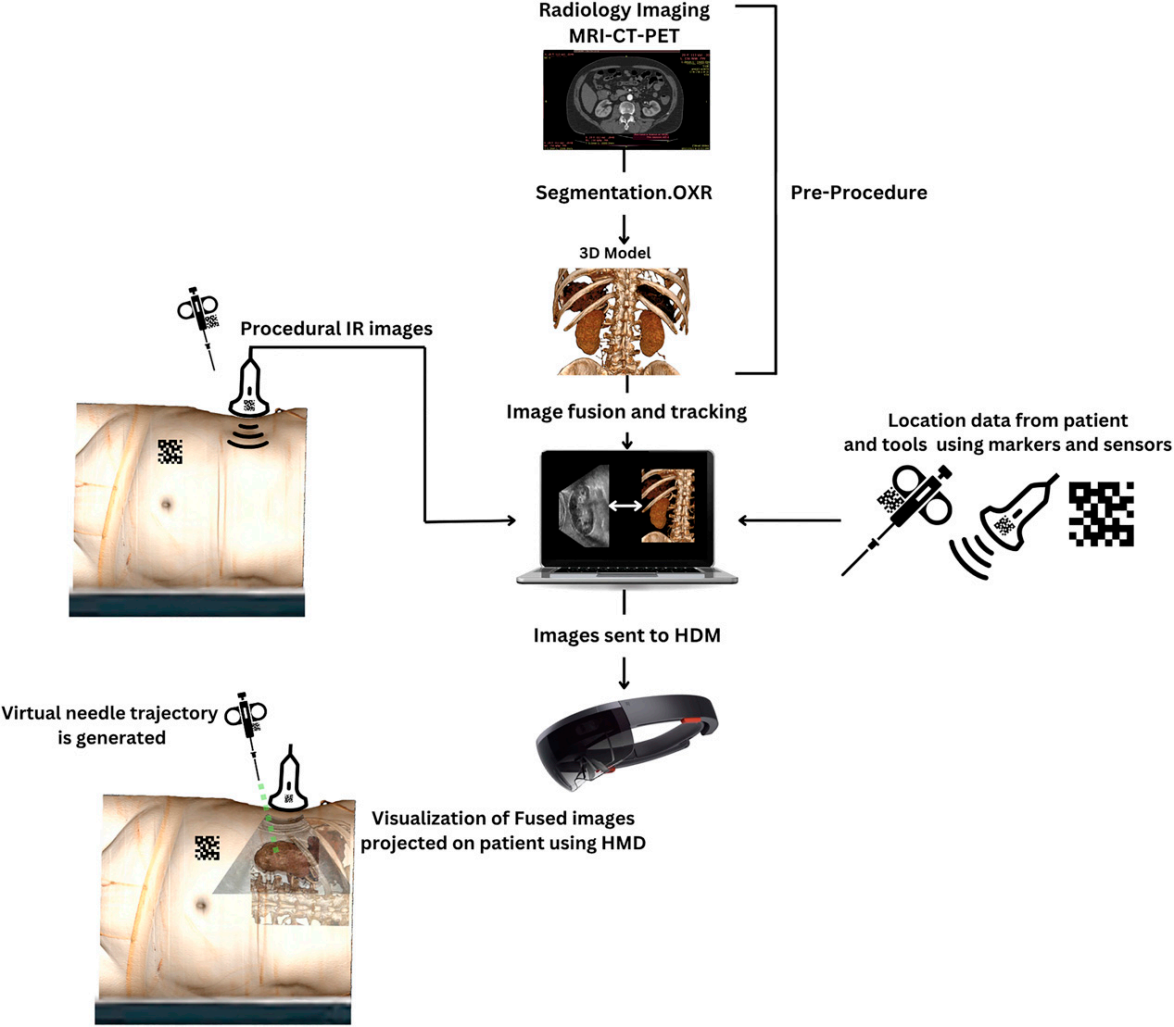
Simplified schematic representation of the components of an AR-assisted operative environment. In this example, pre-procedural CT images are segmented, generating a 3D Model. The companion software combines real-time Ultrasound data and location coordinates of the patient and procedural tool with the pre-procedural 3D model to generate and align fusion images. Data is then transferred to the HMD which shows a 3D hologram superimposed on real structures aligned with real time ultrasound images. A virtual needle trajectory is also generated, which assists in targeting the lesion. 3D, three-dimensional; AR, augmented reality; CT, computed tomography; HMD, head-mounted display.

These components can be fully or partially organized as parts of a more comprehensive AR navigation system/solution (Fig. 2). These systems are either commercially available or developed by individual investigators/researchers. Approximately 28 solutions with various usage in diagnostic and IR are currently approved by the US Food and Drug Administration, including Novarad’s OpenSight (Provo, UT), Medivis’ SurgicalAR (New York, NY), and MediView’s OmnifyXR and XR90 (Cleveland, OH).^14^

**FIG. 3.**
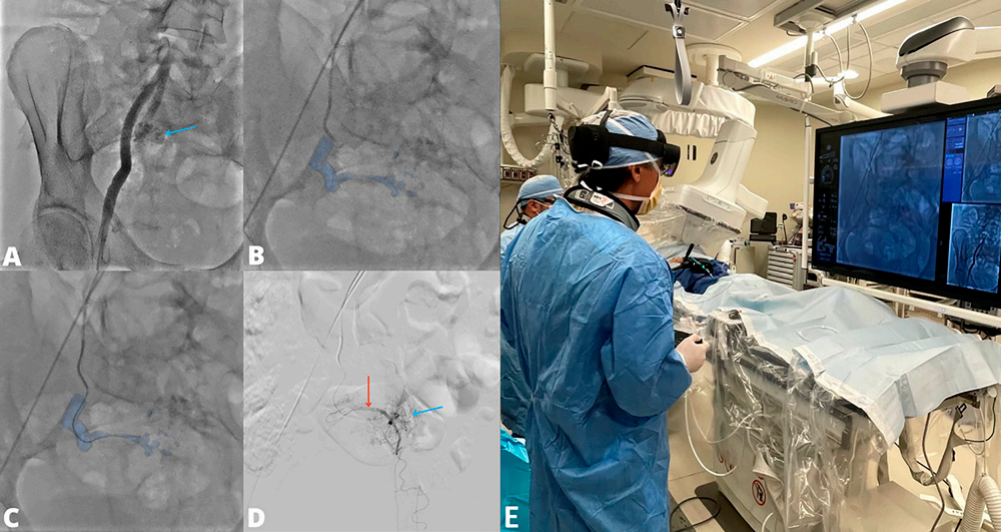
Embolization of a vertebral artery hemangioma with fusion images. **(A)** Right anterior oblique angiographic image showing a right sacral lesion (blue arrow). **(B)** AR 3D visualization of the lateral sacral artery superimposed on fluoroscopic images with the microcatheter advanced into the lateral sacral artery. **(C)** Distal microcatheter navigation within the targeted artery without the use of digital subtracted angiograms helps to reduce the radiation dose. **(D)** Angiographic images showing opacification of the artery (red arrow) and the hemangioma (blue arrow). **(E)** External observer perspective. The radiologist can visualize the fused images through the HMD or by looking at the monitor. This procedure used a robotically steerable catheter with hand-held remote control.

An alternate form of AR system relies on fusion images alone to superimpose preprocedural images on intraprocedural images without the necessary use of virtual displays or holograms (Fig. 3). One example is preprocedural 3D-reconstructed computed tomography angiography or magnetic resonance angiography images superimposed on intraprocedural fluoroscopic images.^15–18^

#### AR displays

**FIG. 4.**
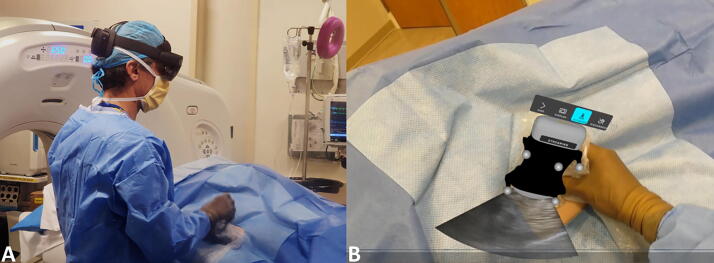
Example of ultrasound image projection along the operative field “through” the patient. **(A)** External observer perspective. **(B)** Radiologist perspective through the HMD.

The primary advantage of an AR display is the ability to place and anchor virtual objects anywhere in space. This is useful for projecting radiological 2D or 3D images and procedural tools onto or “through” the patient (Fig. 4), over the flat panel detector, or inside the computed tomography (CT) gantry.^7,19^

AR displays can be grouped into multiple distinct subtypes, including wearable devices (e.g., HMDs), handheld displays, and stationary displays. These classifications can be further categorized into optical see-through (OST) and video see-through (VST) subtypes. OST displays utilize special transparent lenses that allow for direct views of the external environment while overlaying digital information. VST displays use a video feed (e.g., camera) to indirectly view the external environment. These categories can be further subdivided into monocular or binocular. Monocular displays provide a single viewing channel, while binocular displays simulate depth by providing a separate channel for each eye.^7,8^ Some HMDs support voice control and hand gesture features that enable repositioning, scaling of images, and gain adjustments.^20,21^

**FIG. 5.**
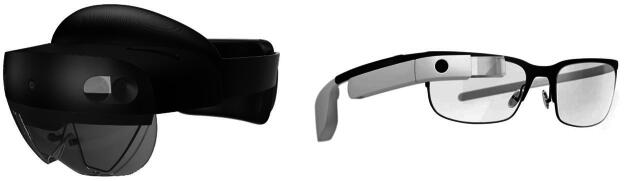
Microsoft’s HoloLens (left) is a type of binocular OST display, while Google Glass (right) is an example of a monocular OST display. OST, optical see-through.

Wearable devices investigated in IR procedures include Microsoft’s HoloLens™ (Redmond, WA) and Brother’s AiRScouter WD-200B (Dublin, Ireland).^2,6,22,23^ Released in 2016, Microsoft HoloLens (Fig. 5) is one of the most used OST-HMDs on the market for image-guided interventions. Several validation studies have reported HoloLens accuracy to be near or within 1 cm.^7^

#### Data processing

Imaging data are processed into virtual objects and displayed to the radiologist. Several steps are required for processing, including segmentation to delimit the area of interest (e.g., liver) and 3D volumetric rendering.^24^ 3D volumetric rendering can be accomplished via surface rendering, which relies on surface meshes at tissue interfaces, or direct volume rendering, which is computationally more intensive but provides the most detailed and accurate visual 3D representation by incorporating the entire data volume.^25,26^ Generated images are then transferred to the AR display for intraoperative visualization.

#### Registration and tracking

Registration refers to the process of precisely aligning virtual objects with the real world so that they appear in the correct position and orientation. Tracking refers to the system’s ability to continuously monitor both user and object movements, updating virtual content accordingly.

Five registration methods are generally used in AR surgical navigation: manual registration, point-based registration, marker-based registration (Fig. 2), surface registration, and calibration-based registration.^8^ Marker-based registration entails the placement of fiducial spot markers (e.g., during preprocedural CT scan for localization in CT coordinates). These are then located with electromagnetic sensors for intraprocedural registration of the segmented anatomy.^27,28^ Fiducial markers can be used to enable motion compensation,^29^ for example, to realign generated images with the patient's chest during breathing.

For tracking, AR devices contain multiple sensors that continuously determine their position within the operative environment, a process known as simultaneous localization and mapping.^13,30^ To improve accuracy, many systems integrate an external optical or electromagnetic tracking system. Calibration of the AR device and external trackers is required and can be performed using manual, semi-automatic, or automatic methods.^7^ Moreover, needles and trocars can be equipped with electromagnetic sensors that enable holographic light rays to be projected from the access tip to the targeted region (Fig. 2).^28^

### Ergonomics and procedural workflow

**FIG. 6.**
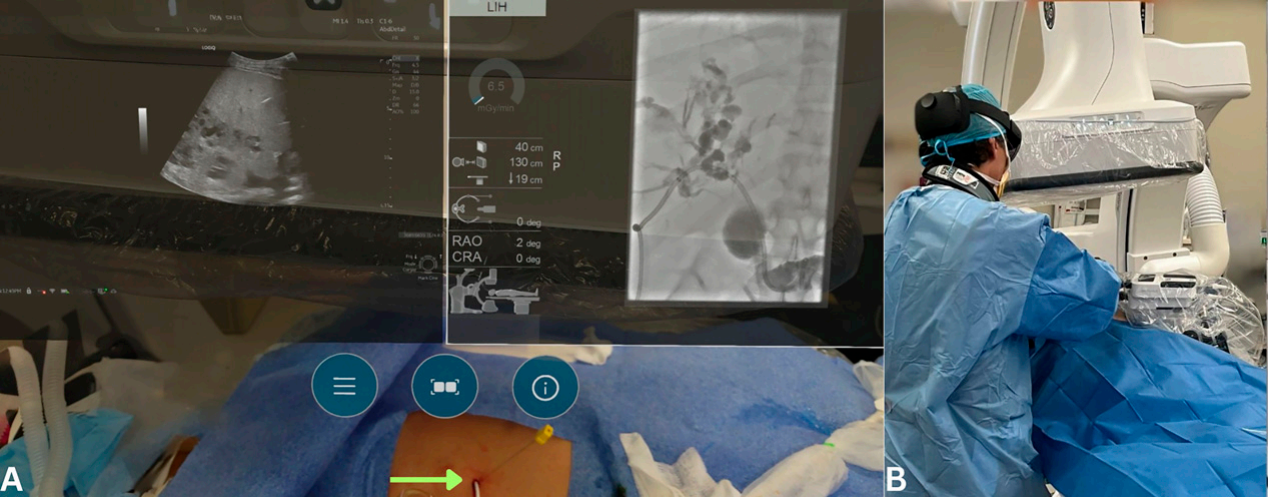
Simultaneous projection of fluoroscopic and ultrasound images during biliary intervention while maintaining the access site (arrow) within the field of view. **(A)** Radiologist perspectives and **(B)** external observer showing the projections are in alignment with the procedural filed, minimizing head turning.

The ability to project both 2D and 3D virtual objects offers several advantages (Fig. 6). AR headsets can deploy virtual 2D monitors anywhere within the IR suite, with the capability to display as many monitors as needed. Real-time streaming of images from devices like the C-Arm or ultrasound (US) machine to the AR headset is possible, and image size can be adjusted as desired, but is ultimately limited by the headset’s field of view.^7,13,31^

The integration of AR headsets enables operators to remain focused on their tasks while minimizing the need to look away from the procedural field, thereby increasing situational awareness by reducing shifts in focus and potentially reducing neck strain.^2,7^ Deib et al. noted that placement of virtual 2D monitors within the procedural field during vertebroplasty enables monitoring of cement placement without diverting attention.^32^ Liao et al. found that augmented reality ultrasound (AR-US) reduced the frequency of visual shift from US display to simulator compared to standard US (1 vs. 5; *p* < 0.001) during central venous cannulation insertion for trainees, suggesting a minimization of neck strain.^33^

Moreover, AR enables direct line of sight with the surrounding environment and allows for interaction with digital objects while maintaining views of the natural world. This provides relevant depth cues and reduces cybersickness. Due to discrepancies between visual and vestibular senses, cybersickness can still occur with AR, but has been found to be less frequent and milder compared to VR.^34^ Solbiati et al. reported no operator cybersickness when using an AR system (Endosight [R.A.W. S.r.l.; Milan, Italy]) for percutaneous thermal ablation of 15 liver tumors.^35^ Despite these benefits, the ability to place and anchor virtual objects anywhere in space can unintentionally preclude visualization of important physical objects, such as instruments and the operator’s hands. Therefore, object occlusion is important for managing virtual content in procedural settings.^36^ Additionally, while human eyes naturally have a wide field of view of about 200° horizontally and 135° vertically, most AR headsets provide less than 90°.^7,37,38^ However, early studies in IR hint toward a general improved spatial perception. Al-Nimer et al. surveyed 10 interventionalists for the usability of the HoloLens platform for 3D holographic percutaneous ablations. Radiologists reported significantly enhanced spatial and depth perception for targeting and avoiding critical structures.^28^

Accurate tracking and registration are critical for image-guided navigation systems. Binocular displays require precise calibration to match the user’s interpupillary distance (IPD). Incorrect IPD can cause poor alignment of the eyes and lenses, leading to image distortion, eye strain, and projection errors due to off-axis viewing.^39,40^ Nevertheless, Solbiati et al. reported no operator eye fatigue when using Endosight for percutaneous thermal ablation of liver tumors.^35^ Accuracy and registration are affected by other factors, including patient motion, breathing, and organ deformation, which are dynamic processes that require dynamic and often computationally intensive solutions.^7^

System latency can also limit the AR system’s ability to track objects in real time. Park et al. described the feasibility of AR headset use on a retrospective case series of three patients undergoing benign solid thyroid nodule radiofrequency (RF) ablation. The reported mean overall system latency was 266 ms. However, the operator did not report noticing a clinically significant intraprocedural lag.^41^

Other considerations include potential discomfort or fatigue from HMD wearing and difficulties in using the devices.^42^ Huang et al. divided 32 novice central line operators into two groups. The first utilized an AR-mounted display (Brother’s AiRScouter WD-200B) while the second employed traditional US for central venous cannulation placement. In a feedback survey conducted among the AR group, over 80% reported that the device did not cause discomfort or fatigue and stated that the information displayed was both relevant and promptly responsive. However, 30% mentioned challenges in adjusting the device, or effects on their procedural skills.^23^ Wu et al. investigated US-guided central venous access using Google Glass (Mountain View, CA). Post-intervention questionnaires found that 75% and 60% of participants had minimal previous exposure to AR and wearable technology, respectively, with 87% of Google Glass users reporting a comfortable experience during the procedure.^43^ Amiras et al. utilized Microsoft’s HoloLens 2™ for CT-guided biopsy training. The research team created a simulated environment using torso phantoms with ChArUco markers for AR tracking. This environment was used to practice biopsies on two retroperitoneal lymph nodes of differing complexities. The study involved junior radiology trainees and four trainers. Feedback collected post-simulation showed that approximately 25% of users found the application user-friendly, while half were neutral, and a minority found usage somewhat difficult. The HoloLens hardware was comfortable for most, with 87.5% reporting no discomfort.^44^ A survey of 10 radiologists by Al-Nimer et al. reported agreement regarding the usability of the HoloLens platform for liver ablation.^28^ Table 1 summarizes the ergonomic advantages and limitations of AR technology.

**Table 1.** Ergonomic Advantages and Limitations of Augmented Reality

Aspect	Advantages	Limitations
Virtual display	– AR headsets can deploy virtual 2D monitors anywhere in the suite, customizable in number and size. – Real-time streaming from devices (e.g., C-arm, ultrasound) enables continuous focus on tasks.	– Limited field of view, typically less than 90°, restricts visualization. – Virtual object placement can obstruct physical objects, instruments, or the operator’s hands.
Object tracking	– Enables accurate image-guided navigation if properly calibrated. – Latency in some setups has been reported as clinically insignificant. – Useful in dynamic environments when accuracy is maintained.	– Sensitive to interpupillary distance misalignment, which leads to distortion and eye strain. – Accuracy affected by dynamic factors like patient motion, breathing, and organ deformation. – System latency may limit real-time tracking.
Situational awareness	– Enhances focus on procedural fields by reducing the need to look away. – Potential reduction in neck strain and increased operator situational awareness.	– Misplaced virtual objects might reduce procedural visibility. – Requires careful management of object occlusion to prevent distractions.
Cybersickness	– Provides relevant depth cues, reducing cybersickness compared to VR.-Minimal discrepancies between visual and vestibular senses.	– Cybersickness, though milder than VR, can still occur.
Headset hardware	– Portable, untethered devices provide flexibility. – Comfortable for short periods, as reported by most users.	– Short battery life (2–3 h) limits use in longer procedures. – Potential for discomfort or fatigue during extended use.
User feedback	– Most users report comfortable experience with AR devices during procedures. – High acceptability and perceived realism in training environments.	– Challenges in adjusting headsets for some users. – Devices can affect procedural skills or require adaptation. – Mixed feedback on user-friendliness, with a minority finding AR somewhat difficult to use.

2D, two-dimensional; AR, augmented reality; VR, virtual reality.

### Applications and outcomes

#### US-guided procedures

A limited number of clinical studies assessed the use of AR technology in US-guided procedures (Table 2), with most of the literature evaluating the use of AR-US on preclinical models, such as phantoms.^23,31,46–48^ Three studies evaluating the use of AR-US in a clinical setting are included in this review. Park et al. described the feasibility of AR headset use on a retrospective case series of three patients undergoing benign solid thyroid nodule RF ablation. Thyroid RF ablation was successfully and uneventfully performed in all three cases, with a mean nodule volume reduction measured at 59.3% ± 18.6 3 months post-procedure.^41^ The reported mean overall system latency was 266 ms ±43, and the operator did not notice a clinically significant intraprocedural lag. Zhang et al. evaluated AR-US for point-of-care US-guided vascular access in pediatric patients (15 successful control and 12 successful interventional). The average procedure completion time was 17.3% shorter using AR guidance with minimal head adjustments. No significant differences were observed regarding the number of puncture attempts and needle redirections.^21^ Al-Abcha et al. used HMDs for AR-US-guided vascular access in 10 patients with six right radial artery and seven right internal jugular vein accesses. The average time to access was 30 s. Access was obtained on the first attempt in all cases, averaging 30 s from lidocaine injection. No complications were reported.^45^

**Table 2.** AR Application and Outcomes for US-Guided Procedures

Study/year	Procedure	Imaging guidance	Equipment	Control	Setting/subjects	Study design	Results (subjective)	Results (objective)
Park et al., 2023^41^	Thyroid ablation	US-AR	HoloLens™ 2, StarMed software, Mirage software	N/A	3 patients, benign thyroid nodules	Feasibility study	No noticeable lag; all procedures successful without complications	Mean nodule volume reduction: 59.3% ± 18.6%.
Zhang et al., 2024^21^	Vascular access	US-AR	Hololens 2, Custom software	Standard US	30 patients (15 control)	Comparative study	N/A	AR-US mean rendering latency of 139.30 ms and rendering frame rate 30 f/s. Average procedure completion time 17.3% shorter with AR-US. Similar puncture attempts and needle redirections. Minimal head adjustment in the interventional group.
Al-Abcha et al., 2023^45^	Vascular access	US-AR	Monocular HMD (Vuzix’s M40°C, Model 509; Rochester, NY)	N/A	10 patients with 13 access (6 redial artery and 7 internal jugular veins	Research Letter/Feasibility Study	No complications	1 attempt was needed in all cases. Average 30 s, 20 ± 8.1 s for arterial, and 35 ± 19 s for venous.

AR, augmented reality; HMD, head-mounted display; US, ultrasound; N/A, not applicable.

#### CT-guided procedures

Studies of AR in CT-guided procedures provide some evidence of improved precision (Table 3) in biopsies and ablations. For biopsies, Albano et al. evaluated an HMD-equipped AR navigation system for CT-guided bone biopsies in eight patients. Preoperative CT images were processed to create 3D reconstructions of anatomical structures, enabling target lesion visualization and needle path planning. Compared to standard CT-guided bone biopsies (*n* = 8), the AR-assisted technique demonstrated significantly fewer CT passes (median 4 [IQR: 4–6] vs. 9 [7.75–11.25]; *p* < 0.001) and lower radiation dose (1034 ± 672 mGy·cm vs. 1954 ± 993 mGy·cm; *p* = 0.021), while maintaining similar procedure durations (22 ± 5 min vs. 23 ± 5 min; *p* = 0.878). Both groups achieved 100% technical success and efficacy without complications.^49^

**Table 3.** AR Applications and Outcomes in CT-Guided Procedures

Study/year	Procedure	Imaging modality	Equipment	Control	Setting/subjects	Study design	Results (subjective)	Results (objective)
Albano et al., 2023^49^	Bone biopsy	CT and AR	Endosight navigation system with HMD (HTC Vive Pro 2; Taoyüan, Taiwan)	Standard CT	16 patients; 8 AR group, 8 control group	Comparative Feasibility Study	No complications observed. Technical success and efficacy both 100%.	AR reduced CT passes (4 vs. 9; *p* < 0.001) and radiation dose (1034 ± 672 mGy*cm vs. 1954 ± 993 mGy*cm; *p* = 0.021). No difference in duration (*p* = 0.878).
Solbiati et al., 2022^35^	Liver tumor ablation	CT and AR	Endosight system with HMD (Lenovo’s Oculus Rift-S; Morrisville, NC)	N/A	8 patients, 15 liver tumors	Feasibility study	No cybersickness or complications reported	High targeting accuracy (3.4 mm, mean); all tumors fully ablated with >90% 5 mm periablational margin.
Gadodia et al., 2022^27^	Ablation of abdominal soft tissue tumors	CT and AR	HoloLens HMD with HoloLens application	Holographic guidance compared to standard imaging guidance	12 patients	Feasibility Study	AR guidance matched standard imaging in 10 cases.	Distance: electromagnetic sensors to AR coordinates = 1.2 mm; anatomical markers to AR = 3.3 mm.
Faiella et al., 2021^50^	Ablation of bone lesions	CT and AR	SIRIO navigation system	Standard CT	193 patients	Comparative Study	N/A	SIRIO reduced procedural time, CT scans, and radiation dose for lesions <2 cm by 25.6 min, 6 scans, and 170.1 mGycm. For lesions >2 cm, reductions were 35.6 min, 5 scans, and 172.7 mGycm. All *p* < 0.005.
Grasso et al., 2021^51^	Lung tumor ablation (RFA/MWA)	CT and AR	SIRIO navigation system	Standard CT	100 patients	Comparative Study	N/A	Lesions ≤2 cm: SIRIO reduced procedural time (32.6 ± 11.8 min vs. 57.2 ± 9 min), CT scans (4.9 ± 2.3 vs. 9.4 ± 2.7), and radiation dose (8 ± 4.5 mSv vs. 14.8 ± 4.5 mSv). Lesions >2 cm: SIRIO reduced procedural time (27.2 ± 13.8 min vs. 47.3 ± 8 min), CT scans (4.7 ± 2 vs. 8.5 ± 2.2), and radiation dose (7 ± 4 mSv vs. 13.3 ± 4.3 mSv). All *p* < 0.001.
Nicolau et al., 2009^52^	Liver tumor ablation	CT and AR	3D CT model with marker-based tracking	N/A	8 patients	Feasibility study	N/A	Accuracy of 4.2 mm during expiratory phases.

AR, augmented reality; HMD, head-mounted display; CT, computed tomography; MRI, magnetic resonance imaging; RFA, radiofrequency ablation; MWA, microwave ablation; N/A, not applicable; 3D, three-dimensional.

While few studies evaluated the use of AR in CT-guided tumor ablation, Solbiati et al. investigated the same HMD-equipped AR system for percutaneous thermal ablation of 15 liver tumors in 8 patients. Pre-procedure CT images were used to segment and reconstruct nodules in 3D, as well as to define probe trajectories. Procedures were guided solely by AR, with probe tip positions confirmed via conventional imaging. Mean system setup time was 14.3 min (12.0–17.2) and mean targeting time was 4.3 min (3.2–5.7). Targeting accuracy averaged 3.4 mm (2.6–4.2), with technical success achieved in all lesions (complete tumor ablation with >90% 5 mm periablational margins). No complications or operator cybersickness were observed.^35^ Gadodia et al. tested AR-guided percutaneous microwave ablation or cryoablation of abdominal soft tissue tumors in 12 patients and found that intraprocedural AR guidance aligned with standard CT imaging in 83% of cases (10 out of 12). In 5 s-stage cases, the mean deviation between AR coordinates and electromagnetic sensors was 1.2 mm, while the deviation between AR coordinates and anatomical markers averaged 3.3 mm.^27^ Faiella et al. compared AR-assisted bone lesion ablations to standard CT-guided techniques in 193 patients. AR significantly (*p* < 0.05) reduced procedural times, CT scans, and radiation doses across both lesion size categories (<2 cm and >2 cm). For lesions <2 cm, the mean differences were 25.6 min, 6 CT scans, and 170.1 mGy·cm. For lesions >2 cm, differences were 35.6 min, 5 CT scans, and 172.7 mGy·cm.^50^ Grasso et al. compared SIRIO-guided (Masmec Biomed; Modugno, Italy) lung thermal ablations to standard CT-guided techniques in 100 patients with malignant lung lesions. The AR system utilized infrared sensors to track needles and displayed their progression on a 3D virtual chest model. The AR group demonstrated significantly reduced procedure times, CT scans, and radiation doses for both lesion size categories (<2 cm and >2 cm) (*p* < 0.001).^51^ Nicolao et al. examined an AR-guided system for liver tumor ablations in eight patients. The method involved placement of radio-opaque markers on the abdomen, followed by CT imaging and 3D reconstruction of anatomical structures. Cameras tracked the needle in real time, guiding insertion by aligning it with the 3D model. The system achieved an average targeting accuracy of 4.2 mm during the expiratory phases.^52^

#### Fluoroscopic percutaneous procedures

Multiple studies evaluated the use of AR in primary fluoroscopically guided procedures and spine-related interventions (Table 4). Auloge et al. compared an AR AI-integrated navigation system with standard fluoroscopy (FL) in percutaneous vertebroplasty for 20 patients with vertebral compression fractures. The AR system utilized fiducial markers for motion compensation and 2D/3D image planning, with virtual needle trajectories displayed on a monitor. The AR group demonstrated superior radiation safety, with significantly lower dose area product (182.6 ± 106.7 mGy·cm^2^ vs. 367.8 ± 184.7 mGy·cm^2^; *p* = 0.025) and reduced fluoroscopy time (5.2 ± 2.6 s vs. 10.4 ± 4.1 s; *p* = 0.005). Despite longer trocar deployment time in the AR group (642 ± 210 s vs. 336 ± 60; *p* = 0.001), both groups achieved 100% technical feasibility and no complications.^29^ Abe et al. assessed an AR-guided percutaneous vertebroplasty system with HMD. Preoperative CT scans generated 3D images superimposed with needle trajectories to guide 10 insertions across five patients. Results showed high accuracy, with insertion angle errors of 2.09° ± 1.3° (axial) and 1.98° ± 1.8° (sagittal). No pedicle breaches or cement leakages were reported.^53^ Wei et al. conducted a randomized controlled trial on AR-assisted percutaneous kyphoplasty versus conventional fluoroscopy in 40 patients. The AR system used HMD for 3D navigation. The AR group demonstrated significantly reduced operation duration (25.12 ± 5.36 min vs. 45.14 ± 3.86 min; *p* < 0.05) and fluoroscopy usage (31.87 ± 9.77 vs. 76.94 ± 8.65; *p* < 0.05). The AR group had lower Visual Analog Scale (VAS) and Oswestry Disability Index scores on 1-year follow up (*p* < 0.05).^54^ Gibby et al. utilized an AR navigation system for 18 image-guided spinal procedures performed on 10 patients, comparing results with phantom control data. The system employed 2D/3D navigational images generated from DICOM files and displayed on a HoloLens. Clinical procedures achieved an 82.35% success rate without realignment, and a mean needle-to-target error of 1.73 mm. No significant differences were observed between phantom and clinical data sets (*p* = 0.121).^55^ Hu et al. compared AR-assisted percutaneous vertebroplasty using marker-based registration and intraoperative image merging to conventional methods in 18 patients. The AR group had significantly lower fluoroscopy usage (6 vs. 18; *p* < 0.001) and shorter procedural times (78 vs. 205 s; *p* < 0.001). Accuracy was enhanced, with a higher proportion of “good” entry points on lateral (81.8% vs. 30.0%; *p* = 0.028) and anteroposterior views (72.7% vs. 20.0%; *p* = 0.020). No complications occurred.^56^ Sag et al. retrospectively analyzed sacroplasty procedures using AR-CT-fluoroscopic guidance compared to standard CT guidance. The AR group showed trends toward reduced radiation dose, total anesthesia time, and cement volume, though differences were not statistically significant (*p* > 0.05). Pain relief, measured by VAS score, was comparable between groups (average reduction: 6.14 vs. 5.25 points; *p* = 0.46).^57^ Jang et al. evaluated AR for transforaminal epidural injections, comparing AR navigation to conventional C-arm fluoroscopy. Preoperative CT images were used for 3D navigation, with marker-based tracking ensuring alignment. The AR group demonstrated significantly reduced procedural time (58.57 ± 33.31 s vs. 124.91 ± 41.14 s; *p* < 0.001) and C-arm usage (3.79 ± 1.97 vs. 8.86 ± 3.94; *p* < 0.001). All procedures were successful without complications.^58^ Finally, Berger et al. presented a case report of AR-fluoroscopy for trigeminal neuralgia rhizotomy, reporting pain relief and no complications.^59^

**Table 4.** AR Applications and Outcomes in Fluoroscopic and Spine Procedures

Study/year	Procedure	Imaging modality	Equipment	Control	Setting/subjects	Study design	Results
Auloge et al., 2020^29^	Vertebroplasty	CB-CT and AR	AR system by Philips Healthcare (Andover, MA)	Conventional FL	20 patients with vertebral compression fractures	Pilot randomized clinical trial	100% technical feasibility; no complications/AR reduced fluoroscopy time (5.2 ± 2.6 s vs. 10.4 ± 4.1 s; *p* = 0.005) and dose-area product (182.6 ± 106.7 vs. 367.8 ± 184.7 mGy·cm²; *p* = 0.025). Longer trocar deployment time with AR (*p* = 0.001). Similar accuracy *p* > 0.05.
Abe et al., 2013^53^	Vertebroplasty	CT, FL, and AR	ARToolKit software with HMD (Moverio, Epson)	N/A	5 patients	Technical note	No pedicle breach or PMMA leakage/mean error in insertion angle: 2.09° ± 1.3° (axial) and 1.98° ± 1.8° (sagittal).
Wei et al., 2019^54^	Kyphoplasty	FL and AR. CT/MR for 3D image generation	Medivi HMD (HoloLens)	Conventional FL	40 patients randomized; 20 AR, 20 control	Randomized controlled trial	AR had lower VAS and ODI scores at 1 year follow-up (*p* < 0.05); greater PMMA injection volume in AR group (*p* < 0.05). AR reduced operation time (25.12 ± 5.36 vs. 45.14 ± 3.86 min; *p* < 0.05) and fluoroscopy times (31.87 ± 9.77 vs. 76.94 ± 8.65; *p* < 0.05).
Gibby et al., 2020^55^	Multiple image-guided spine procedures	CT/MRI and AR	OpenSight AR software with HoloLens	Phantom model control group	18 procedures in 10 patients	Feasibility study	Success rate: 82.35%; mean error to target: 1.73 mm (median: 1.00 mm, SD: 2.20 mm). No difference in clinical and control “target miss” *p* = 0.121.
Hu et al., 2020^56^	Vertebroplasty	CT and AR	Visible Patient software for 3D reconstruction with marker-based registration and projector	Conventional FL	9 patients in AR group, 9 controls	Comparative study	AR improved entry point accuracy on lateral (81.8% vs. 30.0%) and AP views (72.7% vs. 20.0%; *p* < 0.05) / AR reduced fluoroscopy use (6 vs. 18; *p* < 0.001) and procedural time (78 vs. 205 s; *p* < 0.001).
Sag et al., 2022^57^	Sacroplasty	CT-FL and AR	CB-CT-AR software	CB-CT	23 hemisacra; 13 AR, 10 control	Comparative study	No significant difference in VAS score (*p* = 0.46); CBCT-AR trended toward lower dose (*p* = 0.20); no significant differences in anesthesia time or cement volume (*p* > 0.05).
Jang et al., 2024^58^	Transforaminal epidural injections	CT and AR	AR-NAVI system with HMD (HoloLens)	Conventional FL	28 patients randomized (14 AR, 14 control)	Randomized controlled trial	All procedures successful; no complications/AR reduced needle entry time (58.57 ± 33.31 s vs. 124.91 ± 41.14 s; *p* < 0.001) and C-arm usage (3.79 ± 1.97 vs. 8.86 ± 3.94; *p* < 0.001).
Berger et al., 2023^59^	Percutaneous glycerol rhizotomy for trigeminal neuralgia	AR-FL with preprocedural CT	Medivis SurgicalAR with respective AR goggles	Conventional FL	1 patient	Case report	Reports pain relief and no complications.

AR, augmented reality; HMD, head mounted display; CT, computed tomography; MRI, magnetic resonance imaging; CB-CT, cone beam computed tomography; FL, fluoroscopy; NAVI, navigation; VAS, visual analogue scale; ODI, Oswestry disability index; N/A, not applicable; 3D, three-dimensional.

#### Endovascular procedures performed under fluoroscopy

Few studies evaluated the use of AR for angiographic procedures (Table 5). Use of holograms in endovascular procedures was found in phantom/preclinical investigations^64,65^ and only two case reports. Zhu et al. attempted AR-supported inferior vena cava (IVC) filter insertion on a 65-year-old patient without the use of IV contrast. They used preprocedural CT 3D reconstruction data that were projected on the patient via an HMD. The final position of the IVC was confirmed via US.^60^ The same team reported a similar AR-supported procedure on a 48-year-old patient with DVT.^61^

**Table 5.** AR Applications and Outcomes in Endovascular Procedures

Study/year	Procedure	Imaging modality	Equipment	Control	Setting/subjects	Study design	Results (subjective)	Results (objective)
Zhu et al., 2019 and 2021^60,61^	IVC filter placement	AR projecting vascular holograms from pre-operative CT	Non-specified mixed reality headset	N/A	2 patients	Case Reports	Successful	No contrast used.
Schwein et al., 2017^17^	Central venous occlusion (CVO) recanalization	MRV and CB-CT image fusion	Virtual leftline paths overlaid onto fluoroscopy	N/A	4 patients	Feasibility study	Virtual leftlines aligned with guidewire trajectory on fluoroscopy.	Mean fluoroscopy time for lesion crossing: 12.5 ± 3.4 min. Total radiation dose: 15,185 ± 7,747 mGy/m².
Schulz et al., 2016^62^	Endovascular aneurysm repair	CT and CB-CT image fusion	Automatic 3D-3D registration with fusion overlay on fluoroscopy	N/A	101 patients	Retrospective Cohort Study	N/A	Accuracy <5 mm for 68% of patients; caudal deviation occurred in 9% of patients, risking renal artery coverage.
Koutouzi et al., 2017^15^	Thoracic endovascular aortic repair	CT and CB-CT image fusion	Image overlay calibrated onto fluoroscopy	N/A	7 patients	Technical Note	No spinal cord injuries reported.	Image overlay accurately localized intercostal arteries, confirmed by DSA.
Swerdlow et al., 2019^16^	Carotid artery stenting	CT/MRI and FL fusion	2D-3D image fusion with fluoroscopy	Without image fusion	46 patients with image fusion, 70 without	Retrospective Cohort Study	N/A	Reduced radiation dose (207 ± 23 mGy vs. 300 ± 26 mGy; *p* < 0.01); shorter procedure time (47 ± 13 min vs. 54 ± 18 min; *p* = 0.03).
Kaladji et al., 2015^63^	Endovascular aneurysm repair without contrast agent	CT and FL fusion	Preoperative 3D overlay reconstructed and projected onto 2D fluoroscopy	N/A	6 patients	Feasibility study	N/A	Mean error: 1.3 mm (proximal landing zone), 6.5 mm (distal landing zone). No intraoperative endoleaks.

CT, computed tomography; MRI, magnetic resonance imaging; MRV, magnetic resonance venography; CB-CT, cone beam computed tomography; FL, fluoroscopy; N/A not applicable; 3D, three-dimensional; 2D two-dimensional.

Most clinical studies of AR usage in endovascular procedures evaluated overlay or fusion techniques. Schulz et al. evaluated fusion imaging in 101 patients undergoing infrarenal EVAR by overlaying preoperative CT images on intraoperative cone beam CT, achieving <5 mm accuracy in 68% of cases. Nine cases (9% of patients) experienced significant caudal deviations, risking renal artery coverage.^62^ Koutouzi et al. conducted a case series of seven patients undergoing thoracic endovascular aortic repair and used fusion imaging to localize intercostal arteries, avoiding spinal cord injury in all cases as confirmed by digital subtraction angiography.^15^ Swerdlow et al. retrospectively compared 46 patients who underwent carotid stenting with 2D/3D image fusion to 70 patients without it. Image fusion significantly reduced radiation exposure by 31% (207 ± 23 mGy vs. 300 ± 26 mGy; *p* < 0.01), fluoroscopy time by 13% (21 ± 6 min vs. 24 ± 8 min, *p* < 0.02), time to carotid cannulation by 32% (21 ± 9 min vs. 31 ± 8 min; *p* < 0.001), and total procedure time by 13% (47 ± 13 min vs. 54 ± 18 min; *p* = 0.03).^16^ Schwein et al. used image fusion with MR venography in 4 patients with central venous occlusion, achieving a 100% success rate in lesion crossing. The mean fluoroscopy time was 28.8 ± 6.5 min, mean lesion crossing time was 12.5 ± 3.4 min, and mean total radiation dose was 15185 ± 7747 mGy/m^2^.^17^ Kaladji et al. performed EVAR on 6 patients with severe chronic kidney disease using fusion imaging without contrast, achieving a proximal landing zone error of 1.3 mm and a distal landing zone error of 6.5 mm. No intraoperative endoleaks occurred, and renal function remained stable (preoperative eGFR: 17.5 mL/min/1.73 m^2^; 1-week follow-up: 21.7 mL/min/1.73 m^2^; *p* = 0.49; 1-month follow-up: 21.0 mL/min/1.73 m^2^; *p* = 0.28).^63^

## Discussion

AR is currently being applied in a broad range of interventional procedures. The available literature focuses on needle insertion procedures, including various biopsies, ablations, and spine procedures. For vascular studies, the literature focuses on image fusion technologies^15–17,62,63^ with two case reports using HMD-projected holograms. Most studies demonstrated high technical feasibility, with no significant complications reported.^17,29,35,49,53,54^

Main outcomes can be grouped into AR effects on radiation exposure, procedural time, and accuracy. Several studies highlight the potential of AR to reduce radiation exposure. For example, Wei et al. found that AR-guided kyphoplasty reduced fluoroscopy times (*p* < 0.05).^54^ Similarly, Auloge et al. reported a 50% reduction in fluoroscopy time and dose area product during vertebroplasty compared to conventional methods.^29^ Some studies demonstrated faster procedure times with AR. Jang et al. reported significantly reduced needle entry times (*p* < 0.001) during epidural injections^58^ and Wei et al. reported decreased operation time during kyphoplasty (*p* < 0.05).^54^ Conversely, certain studies observed longer deployment times. For example, Auloge et al. (2020) showed that AR usage increased the duration of trocar placement.^29^ This variability suggests that AR may introduce delays in some procedures due to system calibration and setup. Regarding accuracy, AR systems are showing early, promising results. For example, Solbiati et al. reported an average targeting accuracy of 3.4 mm during liver tumor ablation^35^ and Hu et al. showed that using AR in vertebroplasty significantly improved entry point accuracy on both lateral and anteroposterior views compared to conventional fluoroscopy (*p* < 0.05).^56^ In terms of safety, most studies reported minimal adverse events with AR systems. For instance, Abe et al. observed no pedicle breaches or polymethylmethacrylate (PMMA) leakage during vertebroplasty.^53^ However, Schulz et al. noted caudal deviation in 9% of patients during endovascular aneurysm repair, risking renal artery coverage if not corrected.^62^ These results indicate variability between the types of AR systems used and emphasize the persistent need for position confirmation using intraoperative imaging.

While large and multicenter studies regarding ergonomics are lacking, the available literature suggests that AR usage reduces cybersickness and eye fatigue,^35^ decreases focus shift and neck strain,^33^ and improves spatial and depth perception.^28^ Most HMD users also reported usability without discomfort or fatigue^23,28,43,44^ (Table 1). However, AR usage is still limited by technical challenges such as possible narrow field of view, motion-related inaccuracies, and potential usability issues—including object occlusion, short battery life, and user variability^1,7,23,37,66^ (Table 1). To address current AR limitations in IR, future systems should expand the field of view through improved optics, enhance navigation using, for example, AI-based solutions,^30,67^ and extend battery life via efficient power systems. Depth sensing can reduce occlusion,^68^ while customizable interfaces and ergonomic designs can help accommodate user variability. Large clinical studies with rigorous parameters are still needed for a full assessment of the ergonomic advantages and disadvantages of AR in IR.

Persistent limitations necessitate further evaluation before large-scale adoption of AR can occur. Most scholarships are feasibility studies with small sample sizes, providing limited statistical power and generalizability. Preclinical models, such as phantom studies and animal trials, do not accurately replicate the complexities of human anatomy and clinical scenarios. Additionally, the lack of standardized metrics for evaluating AR systems complicates cross-study comparisons. Moreover, cost concerns could hinder the adoption of AR in an already financially strained medical sector. These limitations necessitate a more collaborative approach to AR development and implementation in IR, addressing the current fragmentation across feasibility studies, preclinical models, and evaluation metrics.

## Future Directions and Challenges

Current literature offers insights into the potential benefits of evolving AR technology use in the IR suite. Phantoms and animal studies demonstrate the potential of holograms to minimize the need for radiation exposure and contrast injection in complex procedures. Kuhlemann et al. developed an AR system that provides a 3D holographic view of the vascular system on a vascular phantom for endovascular navigation without the need for radiation exposure. The team used magnetic tracking systems to follow the position and orientation of a catheter inside the vessels.^64^ Yang et al. developed and evaluated an AR-based navigation system for guiding portal vein punctures during transjugular intrahepatic portosystemic shunt creation. The system integrated preoperative CT data with intraoperative electromagnetic tracking. The success rate of AR-guided portal vein access was 100% in both the liver phantom and canine subjects without the need for intraoperative radiation exposure.^65^ Interestingly, a few case reports also demonstrate the early feasibility of holographic guidance. Al-Nimer et al. used AR-projected 3D holograms for percutaneous thermal ablation of liver metastasis in a 64-year-old breast cancer patient. The team used preprocedural multiphasic CT scans to generate the 3D holographic images that were projected onto the patient via HMD. The trocar was equipped with an electromagnetic sensor coil so that a holographic light ray could be projected from its tip to the targeted tumor, allowing targeting without the need for live imaging. Still in development, the holograms were used prior to percutaneous access and after the trocar was advanced to the targeted location.^28^ Similar attempts were made by Zhu et al. for IVC filter insertion.^60,61^

While not always categorized as augmented reality, tools such as EmboASSIST (Siemens Healthineers), which provide virtual injection–based 3D roadmapping, also represent important navigation adjuncts for endovascular procedures and deserve mention in this context.^69^

Although these techniques are still in their infancy, their potential to eliminate radiation exposure and contrast would be of great benefit to patients. These advances hint toward a future where intra-procedure image guidance may be significantly reduced, potentially leading to a paradigm shift in the traditional domain of IR and possibly shifting procedures currently exclusive to interventional radiologists toward surgeons or other specialists. Furthermore, overreliance on AR systems could erode critical procedural skills, such as image interpretation and adaptability in response to unforeseen intraoperative challenges.

## Conclusion

In conclusion, AR offers various applications in IR, with the potential to improve procedural outcomes and ergonomics. The clinical literature suggests a potential reduction in radiation exposure, comparable procedural accuracy, and improved time efficiency. The literature also suggests ergonomic benefits, such as minimized focus shifts and reduced eye fatigue and cybersickness. Despite these outcomes, challenges remain, including the need for rigorous validation, standardized evaluation metrics, and solutions to address high costs. Future considerations must focus on large-scale studies, refinement of AR systems, and strategies to preserve critical procedural skills while ensuring that the integration of AR complements rather than disrupts IR practice.

## Abbreviations Used

2D: Two-dimensional
3D: Three-dimensional
AR: Augmented reality
AR-US: Augmented reality ultrasound
CB-CT: Cone beam computed tomography
FL: Fluoroscopy
HMD: Head-mounted display
IPD: Interpupillary distance
IR: Interventional radiology
IVC: Inferior vena cava
MR: Mixed reality
OST: Optical see-through
RF: Radiofrequency
US: Ultrasound
VR: Virtual reality
VST: Video see-through
